# A Point in Mental Cases

**Published:** 1912-03-09

**Authors:** 


### A POINT IN MENTAL CASES.

### Continuous Administration of Sulphonal.

An interesting account of the effects of the continuous administration of sulphonal is given by Dr. G. M. Robertson in the *Journal of Mental Science*. For some time after its introduction it was believed to be harmless, and for that reason it was much used in the practice of alienists. As ill-effects gradually were noticed after its use over extended periods, especially when large doses were employed, a revulsion of feeling set in, and the drug was practically banished from the pharmacopœias of the mental hospitals. Nowadays it would seem to have regained a measure of its lost popularity, and is used fairly extensively again, though with due precautions as to method of administration and after careful examination of the patient. One of the dangers of the drug arises from its insolubility, especially when the variety used is of coarse grain. Dangers of accumulation can, however, be overcome by administering the drug in the dissolved state. Its solubility is about 2 grains to the ounce at body temperature, so that when 20 grains are to be taken at least half a pint of fluid must be given. It can be prescribed suspended in mucilage. It should not be given to persons with albuminuria, for it is a kidney irritant, and is always excreted slowly. The drug occasionally produces haematoporphyrinuria if administered for long periods, so that it should never be taken for a long time without a break. It is good practice to administer it for six days in succession and then withhold it for three or four days. As to dosage, many hold that not more than 60 grains should be given in any one day, and not more than 40 grains for a man or 30 for a woman if it is to be used for several days. Cases of idiosyncrasy are met with, in which even small doses prove poisonous. It is well, therefore, to begin in all cases with a small dose, such as ten grains. The urine of patients should be examined spectroscopically for hæmatoporphyrin, and if present the drug should be stopped, purgation diuresis and the administration of alkalies instituted at once. In addition to albuminuria and kidney disease, persons suffering from chronic constipation, from melancholia with sluggish bowel action, from stomach or bowel disorders, and from liver diseases should not be given the drug. Chronic alcoholism, anæmia, heart disease, debility, and old age are also contraindications. In the author's opinion it is a dangerous drug except in skilled and careful hands.

